# A Call for Contextualization: Is Cardiopulmonary Exercise Testing the Gold Standard for Exercise Capacity?

**DOI:** 10.7759/cureus.106720

**Published:** 2026-04-09

**Authors:** Sebastian Sitko, Rafel Cirer-Sastre, Isaac López-Laval, Cristina Barrajón

**Affiliations:** 1 Department of Physiatry and Nursing, University of Zaragoza, Zaragoza, ESP; 2 National Institute of Physical Education of Catalonia, Universitat de Lleida, Lleida, ESP; 3 Department of Nursery, Hospital de San Juan de Alicante, Alicante, ESP

**Keywords:** cardiopulmonary exercise testing (cpet), endurance exercise, endurance training, maximal aerobic capacity (vo2max), vo2max

## Abstract

Cardiopulmonary exercise testing (CPET) is widely regarded as the gold standard for assessing exercise capacity. However, this designation is increasingly challenged by both conceptual and practical limitations. CPET outcomes are highly protocol-dependent and obtained under controlled laboratory conditions that fail to replicate the biomechanics, environmental demands, and motivational context of real competition. In addition, its reliance on specialized equipment, technical expertise, and laboratory infrastructure imposes economic and logistical constraints that limit accessibility and scalability. In contrast, advances in wearable technology have enabled the routine collection of field-based performance data, including power output, race results, and power-duration relationships. These metrics provide a direct, ecologically valid assessment of performance, inherently integrating the effects of fatigue, environmental variability, and task-specific demands. As such, they may offer a more relevant characterization of exercise capacity in athletic populations. This perspective argues that field-derived performance metrics should be considered a viable alternative to CPET for assessing exercise capacity in sport settings. We propose that rather than relying on laboratory-based proxies, greater emphasis should be placed on direct measurements of performance obtained in real-world conditions. Such a shift may improve the ecological validity, accessibility, and practical relevance of athlete evaluation in modern sports science and coaching.

## Editorial

Cardiopulmonary exercise testing (CPET) has long been considered the gold standard for evaluating exercise capacity. Its ability to quantify oxygen uptake, carbon dioxide production, and ventilatory thresholds provides detailed mechanistic insights into the physiological response to exercise. Despite its precision and the depth of information it provides, the application of CPET in athletic settings faces important conceptual and practical limitations. Laboratory-based assessments often fail to capture the dynamic and context-dependent nature of real-world performance, which is influenced by factors such as environmental conditions, task-specific demands, and motivational context. In this context, it is crucial to distinguish between physiological exercise capacity (the biological ceiling of an individual), functional capacity (the ability to perform sport-specific tasks), and actual sport performance (the complex outcome of physiological, tactical, and environmental factors). While CPET excels at quantifying the former, it often lacks the resolution to capture the latter two.

One notable limitation of CPET is its strong protocol dependence. While the standardized environment of a laboratory is indispensable for isolating physiological mechanisms and ensuring clinical safety, its inherent detachment from competitive reality may produce partial performance assessments. Outcomes can vary considerably depending on test design, including stage duration, intensity increments, and the type of ergometer. For example, when measuring the maximal aerobic capacity (VO₂max), research shows that only 40%-60% of individuals actually reach a true VO₂ plateau, even when exercising to voluntary exhaustion [1]. In the majority of cases, VO₂peak is reported as a proxy for VO₂max, which can be misleading, as supramaximal verification bouts often yield higher values, though these require additional, more complex procedures rarely performed in practice [2]. At lower intensities, CPET is often used to estimate lactate and ventilatory thresholds. However, attempting to define a metabolic steady state from a continuously increasing workload is inherently flawed, as physiological parameters never stabilize [3]. Together, these limitations highlight that CPET, as commonly applied, may systematically misrepresent both maximal and submaximal exercise capacity, with direct implications for training prescription and athlete assessment.

By definition, CPET is performed under highly controlled laboratory conditions, which inherently fail to capture the complexity of real-world performance. Laboratory tests cannot reproduce the psychological pressures of competition, the environmental variability of outdoor conditions, or the task-specific biomechanics that occur during sport-specific movements. Motivation and effort in the laboratory may differ substantially from those in actual races, where adrenaline, tactical decisions, and competitive context influence physiological responses. As a result, CPET outcomes, while insightful under standardized conditions, often do not reflect an athlete’s true functional capacity in the environments that actually determine performance. This discrepancy raises questions about the ecological validity of laboratory-derived metrics and highlights the need for complementary or alternative assessments that integrate real-world stressors, fatigue patterns, and performance variability inherent to competitive sport.

The practical challenges associated with CPET further limit its applicability beyond specialized research or clinical settings. The requirement for costly specialized equipment, highly trained personnel, and dedicated laboratory space imposes significant economic and logistical burdens, making the routine assessment of large groups of athletes impractical. Even when laboratories are available, the time and resources required for a single comprehensive test, especially if verification protocols or threshold adjustments are included, can be prohibitive. As a result, many athletes and teams are forced to compromise on test completeness or frequency, potentially undermining the accuracy and usefulness of the data. This gap between the highly controlled but resource-intensive laboratory insights and the practical needs of coaches, athletes, and sports scientists highlights a critical disconnect: CPET provides reliable physiological measurements, but these metrics often fail to deliver ecologically valid, directly actionable information that can guide training, monitor progress, or inform competition strategies in real-world settings. Consequently, reliance on CPET alone risks emphasizing laboratory-derived proxies over the performance outcomes that truly matter in sport.

In recent years, advances in wearable technology have facilitated the routine collection of field-based performance data. Devices such as power meters, GPS sensors, and accelerometers allow for the monitoring of variables such as power output, speed, and distance over prolonged periods in real-world conditions. These metrics integrate the effects of fatigue, environmental variability, and task-specific demands, offering a more direct assessment of performance [4,5]. In sports such as professional cycling, marathon running, and triathlon, where power meters and high-frequency GPS data are ubiquitous, field-derived metrics such as the power-duration relationship or critical speed offer a high-resolution alternative to laboratory thresholds. By capturing the conditions and contexts in which athletes actually compete, field-based data provide an ecologically valid perspective that complements, and in some cases may surpass, laboratory assessments.

Given these considerations, it is increasingly important to contextualize CPET within the broader landscape of performance evaluation. While CPET offers valuable mechanistic information, reliance on it as the singular reference for exercise capacity may be misleading. Field-derived performance metrics provide a viable and practical alternative, particularly in athletic populations where the ultimate goal is to optimize competition performance. A hybrid approach, integrating laboratory insights with real-world measurements, may offer the most comprehensive evaluation of exercise capacity and functional potential.

In conclusion, rather than viewing CPET and field-based metrics as opposing methodologies, they should be integrated into a complementary framework. CPET remains the benchmark for mechanistic physiological profiling, but field-derived data provide the necessary ecological context. A hybrid approach ensures that the evaluation of an athlete is both scientifically robust and practically relevant, bridging the gap between laboratory insights and podium outcomes.
